# Analysis of TMEM174 gene expression in various renal cancer types by RNA *in situ* hybridization

**DOI:** 10.3892/ol.2014.2393

**Published:** 2014-07-30

**Authors:** XIUJUN ZHANG, FEN HU, LIJUN MENG, LIXIA GOU, MENGMENG LUO

**Affiliations:** 1 College of Life Sciences, Hebei United University, Tangshan, Hebei 063000, P.R. China; 2 College of Psychology, Hebei United University, Tangshan, Hebei 063000, P.R. China; 3 Department of Environment and Chemical Engineering, Tangshan College, Tangshan, Hebei 063000, P.R. China

**Keywords:** transmembrane protein 174, RNA *in situ* hybridization, renal cancer

## Abstract

Transmembrane protein 174 (TMEM174) mRNA is easily detectable in human kidney tissues and activates AP-1 and promotes 293T cell proliferation. In the present study, RNA *in situ* hybridization was used to detect TMEM174 gene expression in various malignant renal cancer and normal renal tissues. The results showed that TMEM174 exhibits differential expression in renal tissues, with a high positive rate of expression in squamous cell carcinoma with necrosis, papillary renal cell carcinoma and transitional cell carcinoma, and a low positive rate of expression in clear cell carcinoma, interstitial nephritis, undifferentiated carcinoma, retroperitoneal metastatic clear cell carcinoma, adrenal gland metastatic clear cell carcinoma, pelvic cavity metastatic chromophobe carcinoma, severe atypical hyperplasia of transitional epithelium and hyperplasia. Extremely weak expression was exhibited in collecting duct carcinoma, Wilms’ tumor, chronic pyelonephritis, acute pyelonephritis, cancer adjacent normal renal tissue and normal renal tissue. In conclusion, the TMEM174 gene exhibited high expression levels in certain renal carcinomas, which may indicate that TMEM174 may have a significant role in the development and progression of these renal carcinomas.

## Introduction

Transmembrane protein 174 (TMEM174) is a type III transmembrane protein that lacks a clear signal peptide. The N and C terminals are located inside of the cell. TMEM174 was originally identified from a large gene pool by high-throughput cell screening technology. This technique is used for the isolation of functional genes and for the analysis into the mechanisms of gene function (1). Dependent on its transmembrane helices, TMME174 overexpression is able to promote the transcriptional activity of activator protein 1 (AP-1), which is partly mediated by the ERK pathway (2). Our previous study demonstrated high TMEM174 expression in the kidney and also revealed its potential involvement with renal cancer based on its capacity to stimulate cell proliferation (2). The kidney is vital for the maintenance of the salt and water balance within the body, in order to keep a stable internal environment, i.e., for homeostasis. However, the expression of TMEM174 in renal cancer and normal renal tissues remains to be elucidated. In the present study, RNA *in situ* hybridization was used to detect the TMEM174 gene expression in various malignant renal cancer and normal renal tissues. The aim of this study was to provide a theoretical basis for the molecular mechanisms of the development of kidney cancer.

## Materials and methods

### Tissue microarray

Tissue microarrays were purchased from Shaanxi Chao Ying Biotechnology, Ltd., Co. (Xi’an, China). Information on each specimen, consisting of patient age, gender, organization, pathological diagnosis, clinical grade, tumor-node-metastasis classification, clinical stage, specimen type and result information, was available. TNM staging, clinical staging and pathological grading were determined based on the American Joint Committee on Cancer manual (3). Specimens were used from a total of 208 cases, including 178 cases of renal cancer and nephritis, 20 adjacent tissues and 10 normal tissues.

### Preparation of digoxigenin-labeled probes for RNA in situ hybridization

The sense and anti-sense probes that matched the TMEM174 core responding sequence were as follows: Anti-sense, 5′-GAGCATTGTGTTATTATATCAG*AATA GCCTCTAGCGAGGGAGAGAGTATATT-3′DIG and sense, 5′-ATATACTCTCTCCCTCGCTAGAGGC*TATTCTGATA TAATAACACAATGCTCA-3′DIG. The asterisks indicate that the 3′ terminals were labeled with digoxigenin. All probes were synthesized by Shanghai Sangon Biological Engineering Technology and Services Co., Ltd. (Shanghai, China).

### RNA in situ hybridization

Hybridization procedures were performed in this study with the RNA&ISH kit (KD2084, Roche Diagnostics, Indianapolis, IN, USA), based on the manufacturer’s instructions, with certain modifications. The glassware was washed, rinsed in distilled deionized water and autoclaved prior to use. Gloves were worn when the glassware and slides were handled to prevent RNase contamination of the tissue. The hybridization conditions were as follows: Probe concentration, 10 ng/μl; antibody titer, 1:400; washing temperature, room temperarture; dyeing temperature, 37°C; and dyeing time, 2 h. Deparaffinized sections were mounted on Denhardt-coated glass slides (D2532; Sigma Aldrich, St. Louis, MO, USA) and treated with pepsin (0.25 mg/ml in DEPC H_2_O-HCl; Sigma Aldrich) for 30 min in a 37°C water bath. The treated sections were then processed for *in situ* hybridization at 42–47°C for 24 h. The hybridization mixture contained the labeled oligonucleotide probe, 50% formamide, 10 mmol/l Tris-HCl, 1 mmol/l vanadyl-ribonucleoside complex (94740; Sigma Aldrich), 1 mmol/l CTAB (855820; pH 7.0; Sigma Aldrich), 0.15 mol/l Nacl, 1 mmol/l EDTA (pH 7.0), 1X Denhardt’s mixture and 10% dextran sulfate. Subsequent to hybridization, the slides were washed three times, for 30 min each time, in 0.1 mol/l Tris-buffered saline (TBS) at room temperature. The slides were then treated with TBS [100 mmol/l Tris (pH 7.5) and 150 mmol/l NaCl] containing a 1% blocking reagent (Roche Diagnostics, Shanghai, China) and 0.03% Triton X-100 for 30 min at room temperature, and incubated for 30 min with antidioxigenin alkaline phosphatase-conjugated antibodies (Roche Diagnostics) diluted at 1:4,000 in TBS containing 0.03% Triton X-100 and a 1% blocking reagent. Subsequent to being washed three times, for 15 min each time in TBS and 0.05% Tween 20, the slides were rinsed in a diammonimum phosphate (DAP)-buffer [100 mmol/l Tris (pH 9.5) 100 mmol/l NaCl, 50 mmol/l MgCl_2_] and hybridization signals were subsequently visualized using nitroblue tetrazolium and 5-bromo-4-chloro-3-indolyl phosphate as substrates [DAP-buffer (100 mmol/l Tris, pH 9.5, 100 mmol/l NaCl and 50 mmol/l MgCl_2_) in 10% PVA (341584; Sigma Aldrich)].

## Results

### Association between TMEM174 expression and renal pathological cell types

**Figure 1:**
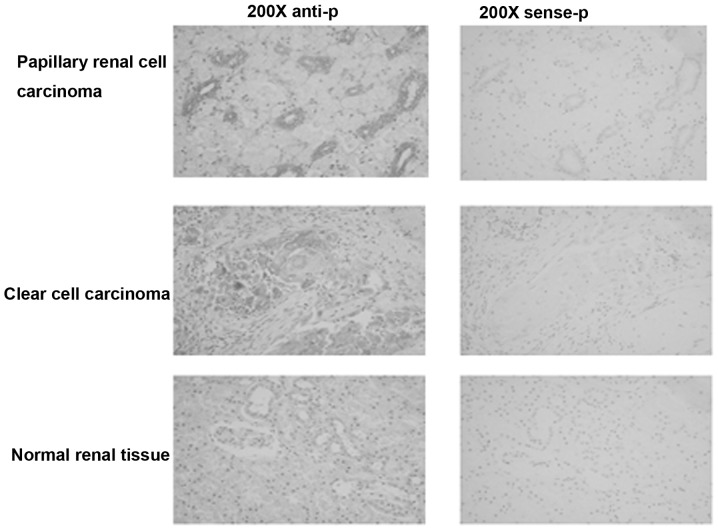
RNA *in situ* hybridization showing expression of TMEM174 in differing tumor tissue samples. The expression of TMEM174 was significantly high in papillary renal cell carcinoma and renal clear cell carcinoma. Extremely weak expression was observed in normal renal tissue. TMEM174, transmembrane protein 174; p, probe.

TMEM174 gene expression in the various malignant renal cancer and normal renal tissues was detected by RNA *in situ* hybridization. As shown in Table I and Fig. 1, TMEM174 exhibits differential expression in renal tissues. TMEM174 had a high positive rate of expression in squamous cell carcinoma with necrosis, papillary renal cell carcinoma and transitional cell carcinoma, and a low positive rate of expression in clear cell carcinoma, interstitial nephritis, undifferentiated carcinoma, retroperitoneal metastatic clear cell carcinoma, adrenal gland metastatic clear cell carcinoma, pelvic cavity metastatic chromophobe carcinoma, severe atypical hyperplasia of transitional epithelium and hyperplasia. Extremely weak expression was observe in collecting duct carcinoma, Wilms’ tumor, chronic pyelonephritis, acute pyelonephritis, cancer adjacent normal renal tissue and normal renal tissue.

### Association between TMEM174 expression and renal pathological clinical stage

The association between TMEM174 expression and renal pathological clinical stage was analyzed. As shown in Table II, the THEM174 expression rate was 58% in phase I tissue samples, 50% in phase II tissue samples, 71% in phase III tissue samples and 50% in phase IV tissue samples.

## Discussion

Each year in the United States, nearly 55,000 individuals are diagnosed with kidney cancer. In order to be effectively treated, an early diagnosis and effective surgical therapy are required. In more advanced-stage cases with involvement of the renal vein and lymph nodes or invasion through the renal cortex, surgical therapy frequently fails (4–8). Kidney cancer cell progression is a coordinated process that comprises cell cycle dysregulation and a specific gene expression program to determine tissue identity (9). The TMEM174 gene is highly expressed in the kidney tissues.

The AP-1 transcription factor is a heterodimeric protein formed from proteins of the c-Fos, ATF, c-Jun and JDP families. In response to a range of stimuli, including cytokines, stress, growth factors and viral and bacterial infections, AP-1 regulates gene expression (10). The cellular transcription factor cAMP-response element binding protein (CREB) binds to specific DNA sequences referred to as cAMP response elements (CREs), thereby regulating downstream gene transcription (11). TMEM174 overexpression has been shown to enhance the transcriptional activity of AP-1 and promote cell proliferation (2). In addition, our recent studies demonstrated that the CREB and AP-1 transcription factors are involved in the transcriptional regulation of the TMME174 gene (12).

When antibodies are not available, RNA *in situ* hybridization is a useful method that allows the determination of the transcriptional expression pattern of a gene (13). With this technique, the expression of multiple RNA species may be assayed using distinct RNA-labeled probes, or the RNA and protein localization within larval tissues may be examined. In the present study, RNA *in situ* hybridization analysis was used to detect the expression of TMEM174, and the results showed that its expression is varies among differing renal tissues. These results indicate that TMEM174 may have a significant role in the development of renal cancer.

## Figures and Tables

**Table I:** Association between TMEM174 expression and renal pathological cell type.

Renal tumors	Number of tumors	TMEM174 mRNA-positive rumors (0–1+/1+/2+/3+), n	TMEM174 mRNA-negative tumors, n
Clear cell carcinoma	59	24 (9/12/3/0)	35
Collecting duct carcinoma	4	0 (0/0/0/0)	4
Squamous cell carcinoma with necrosis	10	9 (0/5/3/1)	1
Wilms’ tumor	20	4 (4/0/0/0)	16
Papillary renal cell carcinoma	20	20 (3/8/9/0)	0
Transitional cell carcinoma	35	32 (5/16/11/0)	3
Undifferentiated carcinoma	4	4 (2/2/0/0)	0
Retroperitoneal metastatic clear cell carcinoma	4	4 (0/4/0/0)	0
Adrenal gland metastatic clear cell carcinoma	4	4 (1/3/0/0)	0
Pelvic cavity metastatic chromophobe carcinoma	2	2 (0/2/0/0)	0
Hyperplasia (sparse renal tubule tissue)	2	2 (0/2/0/0)	0
Severe atypical hyperplasia of transitional epithelium	2	2 (0/2/0/0)	0
Chronic pyelonephritis	2	2 (2/0/0/0)	0
Acute pyelonephritis	2	1 (1/0/0/0)	1
Interstitial nephritis	6	4 (4/0/0/0)	2
Cancer adjacent normal renal tissue	20	10 (10/0/0/0)	10
Normal renal tissue	10	4 (4/0/0/0)	6

Staining was scored using a 0–3+ scale. 0 indicates no staining; 0–1+, indicates trace staining that is weaker than 1+, but stronger than 0. Scores of 1+, 2+ and 3+ indicate an increased intensity of staining. Sub-regions excluding necrosis, macrophages and infiltrated neutrophils and lymphocytes were selected and scored. The intensity score for an array spot is the average of all its sub-regions. TMEM174, transmembrane protein 174.

**Table II:** Association between TMEM174 expression and renal pathological clinical stage.

Clinical stage	n	TMEM174 positive rate, %
I	108	58
II	26	50
III	14	71
IV	4	50

TMEM174, transmembrane protein 174.
